# *The Organon* and Materia Medica Club of the Bay Cities of California

**Published:** 1898-04

**Authors:** Guy E. Manning

**Affiliations:** Secretary


### *THE ORGANON* AND MATERIA MEDICA CLUB OF THE BAY CITIES OF CALIFORNIA.

The regular meeting of the Bay Cities *Organon* and Materia Medica Club was called to order on September 3d, 1897, at 8.45 P. M., by the President, Dr. J. M. Selfridge.

The minutes of August 6th were read and approved. There being no meeting on the 20th there were no minutes of that date.

No other business presenting, a paper was read by Dr. Eleanor Martin on "Psora, as Taught by Hahnemann."

The doctor did not pretend to advance any new ideas regarding it, simply arranging Hahnemann's ideas upon psora, mainly as expressed in *Chronic Diseases*. She spoke of psora as occupying a prominent position in the causation of disease; that it is an internal disease with external manifestations; that infection therefrom was instantaneous, with a period for development. The external manifestation is so often a cutaneous eruption. Sometimes other causes than therapeutic, such as cold, heat, other eruptions, tend to make this disappear, but the internal condition never. The remedy *par excellence* for psora is Sulphur. It has often been given in large doses to the detriment of the case. Only temporarily aided by sulphur baths, producing later the sulphur disease. Sulphur does not cure all conditions of psora, but is usually called for in the beginning, and followed by other remedies. The mode of living, surroundings, occupation, baths, diet, stimulants, all should be looked into and directed. Likewise the mental conditions.

Dr. Selfridge—A difficult thing to verify is whether one dose of Sulphur will cure a case of itch. We seldom see a case of true itch now, or one that has not been tampered with.

Dr. Martin—I have had three cases of true itch well developed in the past few weeks. All recovered under Sulphur.

Dr. Selfridge—Another difficult thing to accept without verification is the cure of syphilis with a single dose of Mercury. (He related a case cured by Mercury, but it was given often, and twice with Syphilinum intercurrently.) I would like to verify with a single dose.

Dr. Martin—How is it a person can have psora and not have the itch, if itch is the manifestation of it?

Dr. Augur—Some of his ancestors must have had the itch.

Dr. Selfridge—Psora is a generic term for a class of diseases. Itch is only one form. At one time psora showed forth as leprosy.

Dr. Augur—I consider itch the father, as it were, of psora—the parent of other diseases.

Dr. Selfridge—I do not so consider it. Psora manifested itself long before itch. (The preface was then read to prove Dr. Selfridge's point, that psora passed through several forms, being before the oldest history of the oldest nation.)

Dr. Selfridge—Itch, then, is a modern manifestation of an old, old disease.

Dr. Martin—The trouble with the whole thing has been in using the term "itch" with "psora," and thus confusing ideas.

Dr. E. Martin—If psora can be cured, why cannot leprosy, the manifestation, be cured?

Dr. Martin—Because leprosy produces a degeneration of the nerve.

This closed the discussion, and the President then appointed Dr. Manning essayist, with "The Cautions of Hahnemann" as his subject for the next meeting.

GUY E. MANNING,
*Secretary.*

